# A Case of Undifferentiated Pleomorphic Sarcoma

**DOI:** 10.7759/cureus.26153

**Published:** 2022-06-21

**Authors:** Reshmi Mathew, Ajay Sethi, Andrew T Flint, Reeba Omman, Jeff House

**Affiliations:** 1 Internal Medicine, University of Florida College of Medicine – Jacksonville, Jacksonville, USA; 2 Pathology, University of Florida College of Medicine – Jacksonville, Jacksonville, USA

**Keywords:** rare soft tissue malignancy, hand mass, pulmonary sarcoidosis, undifferentiated sarcoma, soft tissue tumors

## Abstract

Suspicion for soft tissue malignancy of the hand is usually low because most tumors of the hand are small and benign. We present a case of an elderly female who presented with a rapidly enlarging, ulcerating hand mass over a few months. She was diagnosed with undifferentiated pleomorphic sarcoma (UPS), a high-grade, aggressive soft-tissue sarcoma. Computed tomography (CT) of the chest was conducted for staging purposes. It showed multiple subcentimeter pulmonary nodules, findings that were initially worrisome for metastatic disease but later proved to be newly and incidentally diagnosed granulomatous disease of the lungs. This case highlights the importance of early recognition of potential malignancy in soft-tissue tumors of the hand to facilitate proper referral and initiation of appropriate oncologic therapies. Due to early diagnosis and intervention, our patient had locally advanced disease without metastasis, a type of cancer known to have a high degree of metastatic potential.

## Introduction

Soft tissue sarcomas of the hand are aggressive tumors with a high potential for metastatic disease. UPSs are often a diagnosis of exclusion and account for 10% of soft-tissue sarcomas in adults [1]. These malignancies can affect soft tissues, bone, and retroperitoneum [2]. UPSs occur more commonly in white males, and the incidence of these tumors increases with advanced age, with the highest risk being seen after the sixth decade of life [3]. There must remain a high index of suspicion for malignancy of these soft tissue tumors of the hand because appropriate staging and treatment are imperative to improve oncologic outcomes.

## Case presentation

A 60-year-old African-American female with a past medical history of hypertension presented with a large, ulcerating mass on the dorsal aspect of her left hand. The patient reported that she unintentionally slammed her finger in her car door about two months prior. She reported that the mass first appeared on her finger shortly after this accident and had been rapidly enlarging. She reported only mild pain over the fifth metacarpophalangeal joint of the left hand. She had an associated 60-pound weight loss over the past month. Her only medication included lisinopril, which she recently discontinued due to a new cough she developed. She denied any current or prior tobacco use and alcohol or illicit drug use.

**Figure 1:**
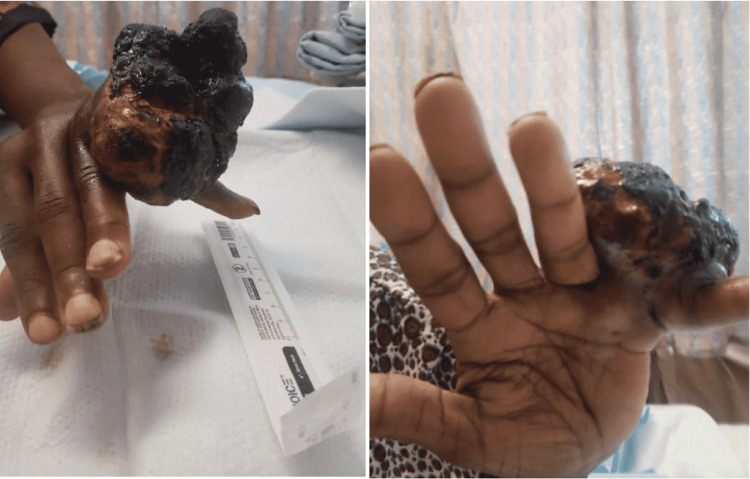
Ulcerating soft-tissue mass (measuring 8.2 x 9.6 x 8.9cm) at the dorsal aspect of the fifth digit, extending into the fourth webspace and abutting the lateral aspect of the fourth digit.

Vital signs on presentation revealed elevated blood pressure (171/96) and tachycardia (heart rate of 106). The rest of her vital signs were unremarkable. Complete blood count was remarkable for iron deficiency anemia and thrombocytosis (platelet count 547,000/cumm). C-reactive protein level was elevated at 135 mg/L (normal range 0.10- 2.8mg/L). The complete metabolic panel was unrevealing. Physical examination revealed the left hand with a large mass protruding from the fourth web space between the fourth and fifth digits (Figure 1).

**Figure 2:**
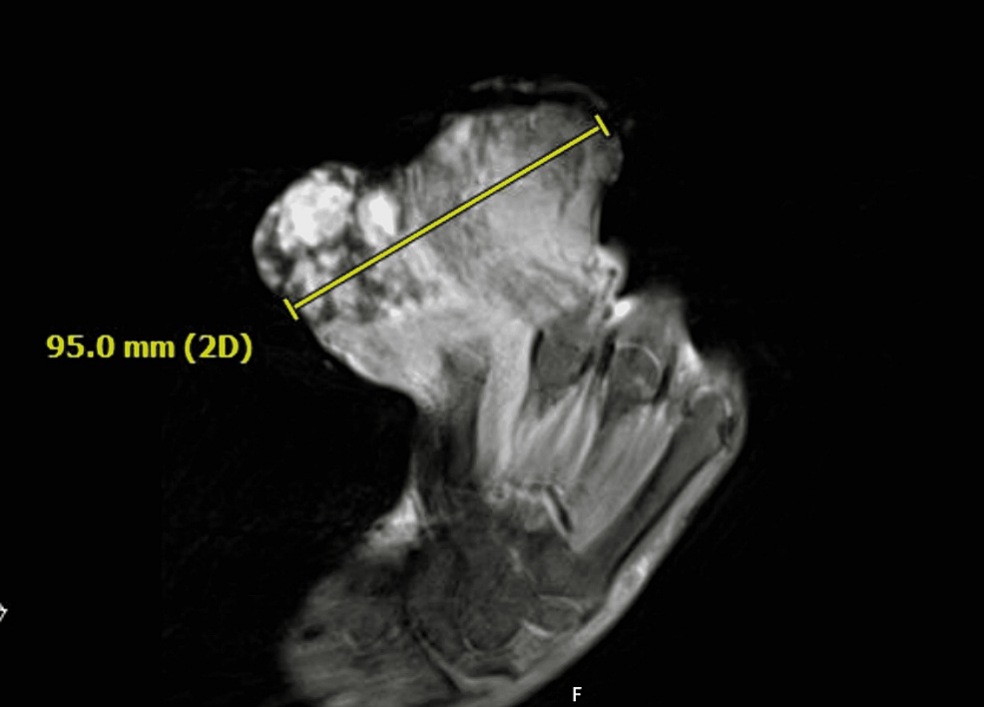
MRI with and without contrast shows a large 9.5cm heterogeneous soft-tissue mass at the dorsal aspect of the fifth digit, with likely involvement of the fifth digit proximal phalanx.

The mass was firm with multiple mounds, and mild bleeding was noted. Sensation remained intact in the median, ulnar, radial, and axillary nerve distributions. Magnetic resonance imaging (MRI) showed a large 9.6 cm heterogeneous soft tissue mass at the dorsal aspect of the fifth digit, abutting the fourth digit (Figure 2).

**Figure 3:**
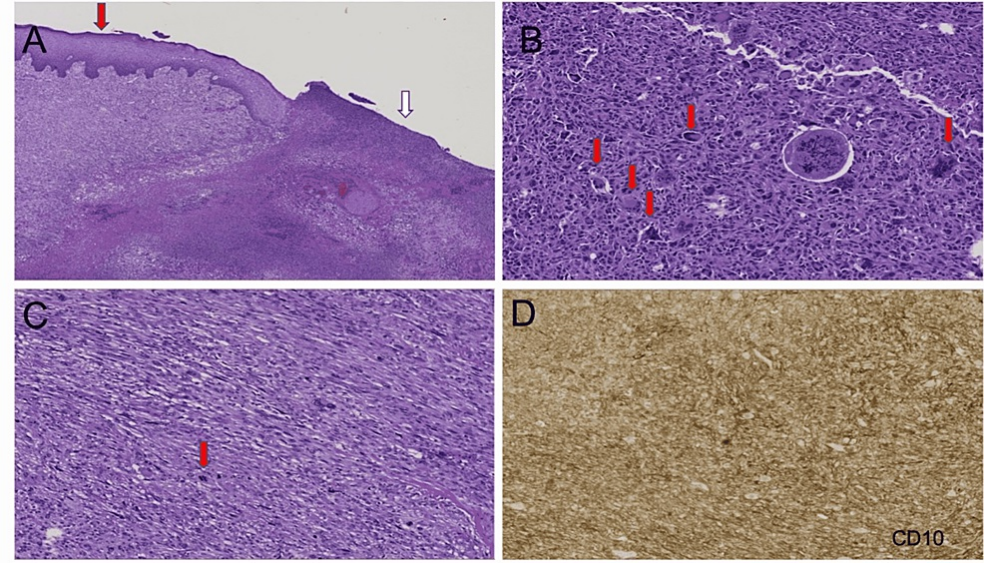
(A) This image shows a lack of epidermal involvement (red arrow) and adjacent cutaneous ulceration (white arrow). (B) This image shows pleomorphic cells with cytologic atypia. There is also the presence of multinucleated giant cells (arrows). (C) This image shows one of many mitoses (arrow). (D) This image shows immunostain positive for CD10.

Magnetic resonance angiography (MRA) showed patent radial and ulnar arteries. There was evidence of vasculature feeding into the mass from the common palmar digital artery branches of the ulnar artery and deep palmar branches of the radial artery. The patient underwent an excisional biopsy of the mass with amputation of the fourth and fifth digits. There was evidence of maggots coming out of the mass during surgical resection. The mass was invading the underlying bone, and Lymphovascular invasion was also identified. Negative margins were achieved per frozen pathology. Pathology of the postsurgical specimen showed high-grade UPS. The immunohistochemical examination revealed cutaneous ulceration (Figure 3A), pleomorphic cells with cytologic atypia, the presence of multinucleated giant cells (Figure 3B), and numerous mitoses (Figure 3C). There was positive staining for CD10 (Figure 3D) and negative staining for AE1/AE3, CD30, CD34, myoglobin, and desmin. Ki-67 expression was more than 90%, further supporting the diagnosis of UPS.

**Figure 4:**
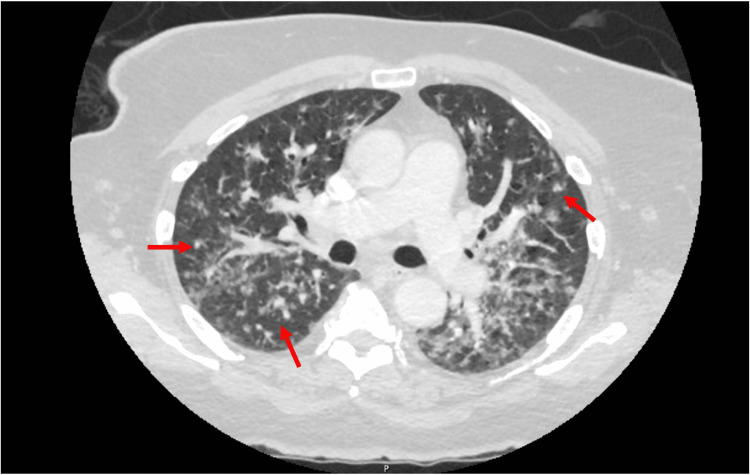
CT chest, abdomen, and pelvis showing multiple subcentimeter pulmonary nodules measuring up to 5mm (arrows) with diffuse tree-in-bud nodularity throughout the lung parenchyma.

Further workup to exclude metastatic disease was initiated. MRI brain with intravenous contrast showed no evidence of metastatic disease, and the whole-body bone scan also showed no metastatic disease. Staging CT of the chest, abdomen, and pelvis had evidence of multiple subcentimeter pulmonary nodules measuring up to five mm with diffuse tree-in-bud nodularity throughout the lung parenchyma (Figure 4).

CT chest findings were initially concerning for metastatic disease. Infectious causes were excluded, including negative Human immunodeficiency virus (HIV) and urine antigens for streptococcus pneumoniae and legionella. The patient underwent bronchoscopy with bronchoalveolar lavage (BAL), which showed evidence of cobblestoning in the right and left upper lobe bronchus. Bronchoalveolar lavage (BAL) cultures, gram stain, and cytology were negative. Endobronchial biopsy revealed non-necrotizing granulomatous changes in the left upper lobe endobronchial lesion. The findings from bronchoscopy and BAL were consistent with a diagnosis of sarcoidosis. Angiotensin-1 converting enzyme (ACE) level was elevated to 111 U/L (normal range 14-82 U/L), and 1,25-vitamin D was elevated to 110 pg/mL (normal range 19.9-79.3 pg/mL), consistent with the diagnosis of sarcoidosis. The patient was started on daily prednisone with recommendations to follow up with pulmonology outpatient for further management. Regarding the patient’s diagnosis of UPS, she was advised to follow up outpatient for the initiation of chemotherapy. However, the patient has not yet followed up due to insurance issues.

## Discussion

Clinical suspicion for soft-tissue malignancy of the hand is usually low because most tumors are small and benign [4]. However, soft-tissue sarcomas of the hand can rapidly enlarge tumors with a high metastatic potential [5]. Clinicians should be aware of specific malignant features of soft-tissue sarcomas, such as size greater than three cm, pain, rapid growth, and subfascial location [5], to expedite proper workup and management. Because of the rarity of malignant tumors of the hand, data on the oncologic outcomes of these tumors remains sparse. Predictions on how these tumors behave and their ultimate outcomes are generally derived from investigations of tumors of the upper extremities [5].

UPSs have no specific line of differentiation and are usually a diagnosis of exclusion [1]. Patients with undifferentiated pleomorphic sarcomas are typically older compared to patients who present with other soft tissue sarcomas [5]. UPSs are frequently found in the limbs, followed by the trunk [1]. In one study investigating the treatment and oncologic outcomes of different soft-tissue sarcomas, undifferentiated pleomorphic sarcoma, synovial sarcomas, and fibrosarcomas had the worst five-year disease survival [5].

Diagnostic workup for soft tissue tumors of the hand should start with conventional radiographs and MRI with and without contrast of the affected hand for local assessment and preoperative planning [5]. CT of the chest should be obtained for malignancy staging purposes. Fluorodeoxyglucose (FDG)- positron emission tomography (PET) can also be included in the diagnostic workup to assess tumor aggressiveness and lymph node metastasis [5]. Immunohistochemical staining remains a vital aspect in diagnosing soft-tissue sarcomas, and a definitive diagnosis of UPS is made by excluding other malignancies with a panel of immunohistochemical markers [6]. Our patient had tumor cells with positive staining for CD10. One study showed that nearly half of all soft tissue sarcomas expressed CD10, and more vital expression was found in high-grade sarcomas [7]. Other soft-tissue sarcomas usually have intense positive staining for AE1 and AE3 [8]. UPS can furthermore be differentiated from other soft-tissue sarcomas by negative staining for AE1/AE3, S-100, and CD34 [8].

One study found that the hand’s metastatic rates for malignant soft tissue sarcomas ranged from 15% to 40% [5]. This patient’s CT chest findings were initially concerning for metastatic disease, especially because UPS is known to have a high degree of metastatic potential. CT chest showed evidence of bilateral diffuse ‘tree and bud’ pulmonary opacities, a hallmark of the distal bronchoalveolar disease. The differentials for these findings include infectious causes such as tuberculosis, nontuberculous mycobacterial lung disease, HIV, or atypical pneumonia. The differential for noninfectious causes includes interstitial lung disease or metastatic disease. Our patient underwent bronchoscopy and was found to have newly diagnosed sarcoidosis, unrelated to her diagnosis of UPS of the hand. Elevated levels of ACE and 1,25 Vitamin D levels further support the diagnosis of sarcoidosis in this patient.

The primary treatment of choice for soft-tissue tumors of the hand is typically limb salvage therapy [5]. However, amputation may be necessary in cases where limb salvage is not feasible or results in less functional outcomes than partial amputation [5]. Overall survival rates for extremity amputation are not superior to limb-sparing surgery [9]. The goal should be to obtain negative margins. Preoperative radiation can be used in patients with tumors larger than five cm or in tumors larger than two cm and located close to neurovascular structures [5]. Postoperative radiation is also an option in patients with postoperative positive margins. Our patient underwent an excision of her hand mass without preoperative radiation therapy.

Additionally, she had negative postoperative margins with no plans for postoperative radiation therapy. The use of adjuvant chemotherapy for patients with resectable soft-tissue sarcoma remains controversial [10]. One study showed minimal efficacy of chemotherapy with respect to recurrence and overall survival [10]. Doxorubicin-only-based chemotherapy regimens were studied, showing no clinical significance in oncologic outcomes. However, Doxorubicin combined with ifosfamide showed further improvement. The decision to manage these tumors should be individualized in each patient, and the risk of toxicity must be weighed against the benefits. Patients with UPS require close monitoring and surveillance. It is recommended that stage II to IV disease be followed every 2 to 6 months for 2 to 3 years, then every six months for two years, and annually after that [2].

## Conclusions

Malignant tumors of the hand are rare, and most of our knowledge on the oncologic outcomes of these tumors is generally derived from cases of tumors of the upper extremities. Because of their rarity, these malignancies can often be mistaken as benign and have unplanned excision without the proper workup beforehand. Performing marginal excision on unsuspected malignant hand lesions may eventually lead to larger resections or amputations being necessary later in the disease course to ensure complete tumor removal with negative margins, leading to worse oncologic outcomes. This case brings awareness to a rare hand tumor known to have a degree of metastatic potential. This case highlights specific features of soft-tissue tumors of the hand that should raise suspicions of malignancy. Early diagnosis and curative excision of UPSs are essential to improve oncologic outcomes. UPSs are rapidly growing with the potential for recurrence and metastasis, making close follow-up and surveillance imperative.
